# Protecting Old-Growth Microbial Communities and Systems

**DOI:** 10.1021/acs.est.3c09835

**Published:** 2024-01-19

**Authors:** Matthias C. Rillig

**Affiliations:** † Freie Universität Berlin, Institut für Biologie, Altensteinstrasse 6, 14195 Berlin, Germany; ‡ Berlin-Brandenburg Institute of Advanced Biodiversity Research, 14195 Berlin, Germany

**Keywords:** microbial communities, ecosystems, biodiversity

Old-growth forests and grasslands^1^ are iconic communities and ecosystems, marked by their unique contributions to biodiversity and ecosystem services. The “old-growth” attribute is central to the efforts justifying their long-term protection. Being “old-growth” is a powerful metaphor in conservation biology and beyond, denoting an ancient, often highly biodiverse community with inherent value. In addition to this important aspect of conservation, the currently unfolding United Nations Decade on Ecosystem Restoration has also highlighted that restoring old-growth systems is exceptionally difficult and that it may take centuries or longer to be successful, for not only forests but also grasslands. Thus, the old-growth status also signals that it will be very challenging to “repair” or recreate such systems once they have been severely damaged.

The term “old growth” has so far been applied to only plant communities in terrestrial ecosystems. The central idea of this paper is that such a status could and should also be extended to microbial systems. The advantages of applying this attribute to microbial systems are immediately obvious. This would deliver a powerful justification for conserving such systems, for valuing them, and it would provide an impetus to direct research efforts at understanding them better and, if need be, restore them. But can such a concept usefully be applied to microbes? At first sight, the very nature of microbes seems to be at odds with this idea. We typically associate microbes and their communities with exceptionally fast growth rates, and microbial communities typically are staggeringly diverse almost everywhere. However, as we discuss below, this is definitely not the case for all systems, as conditions that limit microbial growth are not uncommon.

A useful starting point for exploring this microbial analogue to macroscopic systems is to look for a definition or delineation of old-growth systems in plants, but there is not a universal definition. While there is no clear delineation of old growth,^2^ there are some generally accepted traits of old-growth plant systems, including that they are ancient and generally stable systems representing a successional end point (climax) and they have dead biomass accumulation, often high (and endemic) species diversity, high structural complexity, and slow growth.

The most obvious thought, especially given the realization that most macro-organisms associate with a microbiome, would be to apply this concept to microbial communities associated with old-growth plant communities themselves. Clearly, the microbiome of such systems, for example, in the soil, must have traits that align with the status of the entire ecosystem. This would not be a transformative insight per se, because typically the ecosystem is the focus of protection, and thus, the microbial communities associating with plants in old-growth systems would already indirectly be afforded protection. Thus, the real question is whether there are also microbial candidates that go beyond this mere extension of already recognized old-growth ecosystems.

It seems that there are many examples of slow-growing and potentially ancient microbial communities, structures, and systems that fit this concept. Permafrost soil,^3^ and deep soils, biological soil crusts in arid ecosystems,^4^ or lichen-dominated microbial systems colonizing rock surfaces are some examples from the terrestrial realm. Stromatolites (layered microbial structures involving sediment trapping and binding) are an enigmatic example of a macroscopically visible structure from the aquatic realm, where other examples could be found in deep marine sediment. All of these environments have in common strong limitations on microbial growth, for example, in terms of resource availability (including water or carbon), in terms of abiotic factors such as temperature, or both. As such, they are marked by very slow growth; for example, microbes in the deep Earth crust are estimated to have cell doubling times in the hundreds or thousands of years as a consequence of extreme nutrient limitation.^5^ As an example from a more benign environment, some fairy rings formed by fungi in grassland are likely many hundred years old.^6^ Likely, most of these examples feature accumulation of microbial necromass, in analogy to dead wood accumulation in old-growth forests. Many of these examples are truly ancient or at least very old, and they constitute relatively stable assemblages or features. They all will have a measure of structural complexity, at least at the microbial scale,^4^ which seems a fair criterion to apply. Microbial systems are typically highly diverse, and it is likely that some of that diversity is highly specific to a locale. An example of this could be the fungal growth in the form of wine cellar molds. Following their destruction, such microbial systems are often very difficult to restore; this is, for example, well-known for biological soil crusts^7^ that after disturbance by off-road vehicle traffic take a very long time to recover. It thus appears that specific microbial systems will fit the generally accepted “old-growth” attributes.

What could be the significance of such old-growth microbial systems to environmental science and technology? Highly persistent systems under growth-limiting conditions could harbor microbial diversity and metabolic pathways that are important to the biodegradation of emerging and future pollutants, or they could be home to microbes that can degrade known pollutants but under highly adverse environmental conditions. Given the long-term stability of such systems and their interacting community members, old-growth microbial systems could be a source of interesting biomolecules, for example, for use in medicine or biotechnology. As such, systems persist under often very challenging environmental conditions; studying microbial consortia or (meta)genomes from such old-growth systems could also enhance our understanding of specific biogeochemical cycles and transformation processes.^3,5^ Additionally, could ancient stress-resistant microbial consortia give clues about persistence of microbes outside of Earth? As a consequence of all of these properties, should we explore technological options (biobanks) for the preservation of old-growth microbial consortia *ex situ* if their natural environment is subject to rapid human-caused degradation, to preserve unique genetic resources? A necessary step should be systematic monitoring of old-growth microbial systems to inform protective measures, which could include preservation under artificial conditions as a last resort.

We have in recent decades, notably with the arrival of high-throughput sequencing and metagenomics techniques, learned a lot about microbial systems. It seems on the basis of this knowledge we should afford microbial systems with such specific traits, enigmatic and ancient in their own right, some of the same status, valuation, and protection as their macroscopic cousins. One important step in this direction would be to help protect such microbial communities and systems by broadening the concept of old-growth from the classical plant-focused perspective to also include microbes. Old-growth microbial communities are threatened by human influence, including many factors of human-caused global environmental change,^8^ including chemical pollution, climate change, and potentially invasive microbes. Just like the relatively recent inclusion of grasslands within the “old-growth” concept,^1^ microbial systems similarly stand to profit in terms of conservation policies, research attention, and ecosystem management. In addition, and closely aligned with these goals, such a status would also help instill a sense of wonder about such systems in the general public.
